# Cinchonidia

**Published:** 1877-06-20

**Authors:** 


Cinchonidia.—This article, as manufactured by Powers & Weightman, is now attracting much attention from the profession as a substitute for the sulphate of quinine.

Premium.—See premium notice on first page of advertisements.

